# Self-referrals and associated factors among laboring mothers at Dilla University Referral Hospital, Dilla, Gedeo Zone, Ethiopia: a cross-sectional study

**DOI:** 10.1186/s12905-022-02002-7

**Published:** 2022-10-11

**Authors:** Aneleay Cherinet Eritero, Kahsay Zenebe Gebreslasie, Alem Tadesse Asgedom, Abriham Shiferaw Areba, Aregahegn Wudneh, Yesuneh Bayisa, Wondwosen Molla

**Affiliations:** 1 Department of Midwifery, College of Health Science, Dilla University, Dilla, Ethiopia; 2 Department of Midwifery, College of Health Science, Mekelle University, Mekelle, Ethiopia; 3 School of Public Health, College of Health Science, Dilla University, Dilla, Ethiopia; 4 School of Medicine, Dilla University, Dilla, Ethiopia

**Keywords:** Self-referral (bypassing), Primary health care units, Laboring mothers, Dilla University Referral Hospital

## Abstract

**Background:**

When medical cases are difficult to manage at the level of primary health care units (PHCU), formal referral assists patients transferring to a higher level of care. In contrast, self-referral and bypassing are synonymously used in literature to describe the phenomenon of patients skipping their units to get basic medical services, even though they are close to their residence. Though proper and timely referral prevents the majority of deaths from obstetric complications in developing countries, more than 50% of referrals are self-referral trends. Such patient practice is increasingly becoming a concern for many health-care systems.

**Objective:**

To assess the magnitude of self-referrals and associated factors among laboring mothers at Gedeo Zone, Ethiopia.

**Methods:**

Facility-based cross-sectional study was conducted from August 1-September 30/2021 among laboring mothers at Dilla University Referral Hospital. A systematic random sampling technique was used to select 375 laboring mothers. Data were collected using a face-to-face interview with a structured questionnaire. Data were entered into a computer using Epi-Data 4.6 statistical program and then exported to STATA version 16 for analysis. In bivariate analysis variables with a *p*-value ≤ 0.25 were selected as a candidate variable for the multivariable analysis. *P*-value < 0.05 at 95% confidence interval considered as a statistically significant associations in the multivariable analysis.

**Result:**

375 eligible mothers participated in the study, with a response rate of 98.16%. The magnitude of self-referrals among laboring mothers was 246 (65.6%) with 95% CI (0.60–0.70). Time ≥ 30 min to reach nearby facilities (AOR = 1.74, 95% CI, 1.08, 2.81), having no medicine supplies at nearby facilities (AOR = 1.75, 95% CI, 1.08, 2.82), having no equipment and supplies at nearby facilities (AOR = 1.70, 95% CI, 1.03, 2.78), having ANC visits ˃ 3 times (AOR = 0.29, 95% CI, 0.15, 0.55) and having poor perception of health provider technical competence at nearby facilities (AOR = 2.97, 95% CI, 1.83, 4.79) were found as significant factors for self-referral.

**Conclusion:**

The magnitude of self-referral was high. Frequent Antenatal visits were protective, however time to reach the nearest facilities, perception towards health care providers, medicine, equipment and supplies at the nearest facilities were positive influencing factors. Government stakeholders should keep working on improving the quality of health service, especially at primary health care units(PHCU).

## Introduction

The World Health Organization (WHO) places a strong emphasis on the need for referral systems to operate efficiently and to be integrated with primary care units (PHCU) [1]. Thus, in the effort to reduce maternal and child mortality rates globally, formal referral systems are an essential part of high-quality healthcare systems. The terms "self-referral" and "bypass" are used interchangeably to describe a behavior by patients in which they skip local primary health care units (PHCU) in favor of seeking treatment directly from higher facilities [2, 3]. However, when medical or obstetric conditions are challenging to manage at the primary health care units (PHCU), a formal referral system is used to transfer patients to the next level for better care. Thus, mothers who have obstetric complications are frequently referred to higher facility for cesarean delivery and treatment [4–6].

Ethiopia's healthcare system is structured in a hierarchical manner in a similar fashion to other countries. The health policy outlines that the Primary Health Care Unit (PHCU) comprises health centers, health posts, and primary hospitals [7]; General hospitals on the secondary level; and Specialized hospitals on the tertiary care level. The primary health care unit (PHCU) provides an important role in the link with higher referral facilities. In addition, these facilities function by giving basic emergency obstetric and neonatal care (BEmONC) [6, 8]. However, some studies found a tendency for under-utilization of most peripheral public health facilities, indicating a break in the linkage of the referral system [9, 10].

Self-referral (bypass) to seeking childbirth directly at tertiary hospitals has enormous implications for service delivery as well as human resources within a health system. Thes condition can result in the under-utilization of primary health care units (PHCU) and simultaneous overcrowding of tertiary hospitals, thereby weakening quality service for those needing urgent medical management at referral hospitals [4, 11]. Further, this problem can lead to a waste of resources and the skills of healthcare providers who are expected to provide basic emergency obstetric care (BEmONC) at these facilities. In addition, it may cause excessive costs due to the migration of pregnant women to distant and more expensive centers [12, 13]. According to studies, self-referral is also less effective at detecting high-risk pregnancies than formal referral pathways [2].

The extent of the self-referral trend varies between healthcare settings. Studies from developing nations revealed that self-referral accounts for more than half (50%) of all referral patterns [4]. For instance, African studies showed that the magnitude of self-referral was 60%, 87% and 96.1% in Nigeria, Sudan, and Kirinyaga district, Kenya, respectively [14–16]. Similarly, in studies done in western part of Ethiopia shows, the magnitude of self-referral in general and referral hospitals was identified as more than 82% [10, 17]. Furthermore, studies conducted in north-west and southern Ethiopia shows that 63.9% and 67% of mothers, respectively, avoided their catchment public health centers[18, 19].

According to studies conducted in developing countries, the following factors are important in self-referral(bypass): availability of medicine, equipment, and supplies [20, 21] community awareness of the referral system, readiness of the nearest facility for childbirth, basic emergency obstetric care signal functions, and the availability of free transportation at the nearest facility. Poor perceived quality of care at clinics, as well as quality of care at government dispensaries and health, are also factors that contribute to self-referral(bypass) [10, 22–24].

The bypassing trend in Ethiopia is attributed to a number of factors. According to the reviewed literature, patient demographics, including age, educational status, and financial disparities, as well as facility characteristics such as long travel times to reach nearby health facilities; drug availability; equipment and supplies; parity; antenatal care visits; and client perception of providers are all factors influencing self-referral (bypass) behavior [9, 10, 17, 19, 25].

Despite the Ethiopian government's impressive efforts to ensure that everyone has access to primary health care units (PHCU) [26, 27], a few studies conducted in the country indicate that much work remains to be done in reducing bypassing problems [10, 25]. Furthermore, in the study area, such health care practice with the aforementioned trend contributes more to the problem's burden. Accordingly, this study will be extremely beneficial to health professionals working in primary health care units (PHCUs), as well as stakeholders and policymakers in the health sector, in order to avert the problem and prevent the occurrence of similar future events. To the researchers' knowledge, there are limited studies conducted with respect to maternal health care utilization in the study area and at the national level. Therefore, this study aims to assess the magnitude of self-referral among laboring mothers and factors associated with it at Dilla University Referral Hospital.

## Methods

### Study design, period and setting

A facility-based cross-sectional was conducted from August 1 to September 30, 2021, at Dilla University Referral Hospital. Dilla town is the capital of Gedeo Zone, which is located in the South Nation and Nationalities People Region (SNNPR). It is 360 km from Addis Ababa, Ethiopia's capital city, and 90 km from Hawassa, the regional capital city, and has a longitude and latitude of 6024′ 30′′ N 38° 18′ 30′′ E with an elevation of 1570 m above sea level. The Zone has two city administrations and eight woredas. The referral hospital is one of the busiest facilities in the region, serving the entire Gugi Zone and some parts of the Sidama region. There are about 550 health professionals in the hospital, of which 21 are midwifery professionals and four are obstetricians and gynecologists. The hospital has a total of 53 beds in the Obstetrics & Gynecology ward, of which 4 are delivery coaches. The referral hospital has given around 3475 delivery services in the fiscal year of 2020.

### Source population

All laboring mothers who were admitted to Dilla University Referral (DURH) and found during the data collection period were included.

### Study population

Randomly selected laboring mothers who were admitted to DURH.

### Eligibility criteria

This study included all expectant mothers who were admitted to Dilla University Referral (DURH) during the data collection period. Mothers who received ANC follow-up at the hospital as well as those who are unable to communicate due to serious illness were excluded in this study.

### Sample size calculation and sampling method

The sample size for this study was determined using a single population proportion formula with *P* = 0.5 (50% hypothesized proportion of self-referred mothers in Dilla University Referral Hospital) level of significance 5% (α = 0.05), the margin of error 5% (d = 0.05). The calculated final sample size was 384.

A systematic random sampling technique was used with a K-value of 2. We have used the previous three-month report on the total number of mothers attended at the facility, which was 868, to select a total of 384 laboring mothers for an exit interview. The first study participant was determined randomly.

### Operational definition

*Self-referral*—is defined as when laboring mothers seek delivery service directly from Dilla University Referral Hospital and without being formally referred (not have a referral paper) for a specific obstetric/medical reason. It will be assessed by a question with an expected response of 0 for "No" or 1 for "Yes" (self-referral mothers are whose response was "Yes").

*Distance***—**This was measured from the report of the mother walking hours to the health facility.

This was dichotomized into two categories, “1” if the mothers reported the walking hours to reach the nearby health facility to be > 30 min; otherwise, it was coded “2” for ≤ 30 min [9]**.**

### Mothers perception toward technical competence of the health providers

Good perception—those who scored median and above for perception related questions.

Poor perception—those who scored below the median score for perception related questions. The content items development for the perception related questions were bases on International Confidartion of Midwiferey (ICM) Essential Competencies for Midwifery Practice [28].

### Data collection tool and procedure

The data collection tool was adapted from another study and contextualised to the study setting. For consistency, it was written in English, translated into Amharic and Gedeoofa, and then back-translated into English by an independent translator. The questionnaire obtains information on the main variables that are: 8 socio-demographic, 3 questions on referral status, 11 Likert scale questions on mothers' perception of the technical competence of the health provider, 8 facility characteristic-related questions, and 3 obstetric related questions. Three midwives with BSc degree holders who work outside of the study area and who speak Amharic and Gedeofa were selected for the data collection. On each day, those admitted to the postnatal room who had delivered at Dilla Hospital and meet eligibility criteria were selected to participate in the study. The purpose of the research was also briefly explained by the data collectors with the guidance of the principal investigator. The actual data collection was done by reading the questionnaire to study participants, and the data collectors’ supervision was done daily.

### Data quality control

Before the actual data collection, a pre-test was done on 10% of the total participants in the hospital near the study setting. After the pretest was done, a question that needed language clarity or understandability was revised and adjusted. Then, for data collectors, a one-day training was given on the objective of the study, how to use the data collection tool, and how to maintain confidentiality and the rights of respondents throughout the research process. All the details of the purpose of the research, as well as the necessary information, were given to the study participants. The collected data was checked thoroughly for completeness and consistency daily.

### Data processing and analysis

Upon completion of data collection, the questionnaire was checked for completeness, edited, and coded. Then data are entered into the Epi-data version 4.6. Then, it was exported to STATA version 16 analysis. First, descriptive analysis was done using frequency and percentage, and then presented using text, charts, and tables. To proceed with regression analysis, a Hosmer–Lemeshow goodness of fit test was done, and a good fit for the dataset was found (*P*-value 0.93). The variance inflation factor (VIF) and the tolerance test were used to examine multicollinearity. The result of VIF was less than 2, while the tolerance test was > 0.1. Then, a bivariate analysis was done, and all explanatory variables with a p-value less than 0.25 were included in the multivariable analysis. A multi-variable analysis was employed to determine independent determinant factors among the explanatory variables with an adjusted odds ratio (AOR) with a 95% confidence interval computed. *P*-values less than or equal to 0.05 were used to determine if there was a statistically significant association with the outcome variable.

## Result

The response rate for this study was 98.16% due to seven questionnaires returned partially filled. 375 respondents fully participated in this study recruited by systematic random sampling techniques in the postnatal ward.

### Socio-demographic characteristics for the respondents among laboring mothers at Dilla University Referral Hospital, Dilla, Ethiopia, 2021

The mean age of study subjects was 27.37 years (SD + 4.172), with a minimum of 16 to a maximum of 35 years. The majority of respondents 263 (70.13%) were in the age group of 25–34 years. About 193 (51.47%) of respondents were from rural areas; 182 (48.53%) were urban residences; and 234 (67%) of respondents responded that they attended primary level education (1–8 grade). About 134 (35.7%) of the participants responded that they earn a monthly income of 1001–1500 Ethiopian Birr (ETB) (Table 1).

**Table 1:** Sociodemographic characteristics of the respondents at DURH, Gedeo Zone, SNNPR, Ethiopia, 2021

Socio-demographic characteristics	Frequency	Percentage
*Residence*		
Rural	193	51.5
Urban	182	48.5
*Age group*		
< 25 Years	107	28.5
25–34 Years	263	70.1
> 35Years	5	1.3
*Marital status*		
Single	41	10.9
Married	297	79.2
Divorced	24	6.4
Widowed	13	3.5
*Educational status*		
No education	34	9.1
Primary	235	62.7
Secondary	83	22.1
Collage or above	23	6.1
*Occupational status*		
Farmers	68	18.1
Housewife	173	36.5
Merchants	56	14.9
Employee	93	24.8
Others	21	5.6
*Family size*		
1–3	136	36.3
4–5	189	50.4
> 5	50	13.3
*House ownership*		
Rented	126	33.6
Private/owned	247	65.9
No house	2	0.5
*Mothers’ monthly income*		
< 500 ETB	148	39.5
501–1000 ETB	132	35.0
> 100 ETB	95	25.3

### Respondents referral status and information regarding the referral system, Dilla, SNNPR, Ethiopia, 2021

Among 375 respondents who were enterred in the analysis, 246 (65.6%) were self-referrals(bypasser). Only 150 (40%) said they have information about the nearest health facilities being the first contact in the health care system, while 225 (60%) of the respondents said they don’t have information about the referral system. Concerning the mother's source of information on the referral system where the nearest health facilities are the first contact in the health care system, more than half (55.6%) of the respondents said they got it from health care providers. Due to self-referral, the majority of the respondents 295 (78.7%) reported that they bypassed health centers, and they were followed by 69 (18.4%) primary hospitals and 11 (2.9%) general hospitals.

### Mother’s judgment for the facility-characteristics of the nearest health facility, Dilla, Ethiopia, 2021

The main findings of this section are that approximately 207 (55.2%) mothers reported that it took them 30 min to walk from their home to the closest medical facility.When mothers were asked to rate the quality of the medicine and laboratory services they received from nearby health institutions, around 189 (50.4%) and 209 (55.7%) of the mothers said they did not feel confident in receiving enough medicine and laboratory services. Around 157 (41.9%) of respondents say the nearest health facility labor ward has a shortage of equipment and supplies. 216 mothers (57.6%) said that the nearby health facility lacked both electricity and clean water, which are two more crucial physical elements. About 237 (63.2%) of the mothers said they couldn't receive ambulance services when there were emergency situations (Table 2).

**Table 2:** Mother’s judgment for the nearest health facilities general characteristics, Gedeo zone, SNNPR, Ethiopia, 20,119

Facilities general characteristics	Self-referred		Frequency (%)
	Yes (%)	No (%)	
*Adequate human resource*			
Yes	96	53	149 (39.7)
No	150	76	226 (60.3)
*Laboratory service*			
Yes	104	62	166 (44.3)
No	142	67	209 (55.7)
*Medicine supplies*			
Yes	111	75	186 (49.6)
No	135	54	189 (50.4)
*Equipment and supplies*			
Yes	131	87	218 (58.1)
No	115	42	157 (41.9)
*Clean water and electricity*			
Yes	100	59	159 (42.4)
No	146	70	216 (57.6)
*Adequate private room*			
Yes	99	46	145 (38.7)
No	147	83	230 (61.3)
*Ambulance service*			
Yes	89	49	138 (36.8)
No	157	90	237 (63.2)
*Walking hours to reach the nearest health facilities*			
< 30 Mins	100	68	168 (44.8)
≥ 30 Mins	146	61	7 (55.2)

### Mothers perception towards the health care providers technical competence working at the nearest health facilities, Dilla, Ethiopia 2021

The internal consistency reliability test was done for perception-related Likert scale items by using the popular method of Cronbach's alpha, and it was reported at 0.709, which was around the recommended value of 0.70. Meanwhile, to further test the reliability of the tool when the scale of item deleted was used on the fifth item, a similar result of an alpha value of 0.709 was reported.

When the analysis of 5-point Likert scale perception related data was done, the normality of distribution was assessed by the Kolmogorov–Smirnov test and a *P*-value of 0.000 was reported. That is less than the suggested value of 0.05, which violates the assumption of normality. So, the median value, which is 28, was used to set the cut point to categorise the perception level. That is, poor perceptions were those who scored below 28, while good perceptions were those who scored 28 and above for perception-related questions. During analysis, the responses "strongly agree" and "agree" were classified as "agree", and responses "strongly disagree" and "disagree" were classified as "disagree", and the response "neutral" was left as it is.

Our result among perception-related items shows that 55.2% of respondents have a poor perception for the technical competence of health care providers at the nearest health facility, while the rest, 44.8%, have a good perception. According to 5 point Likert scale items, the respondants lowest mean perception score was recorded for the statement "the health care providers at the nearest health facility have the motivation to help clients," followed by "providers at the nearest health facility show courtesy to clients." While the highest score was for the statement "Health professionals provide timely follow-up during labor," followed by "Health providers at the nearest health facility listen carefully to clients' concerns during labor and delivery." (Table 3).

**Table 3:** Mothers Perception to ward technical competence health providers working at the nearest health facilities, SNNPR, Gedeo zone, Ethiopia, 2021

S No	Questions	Disagree	Neutral	Agree	Mean of perception score
1	Providers at the nearest health facility show Courtesy toward clintes	268 (71.5%)	23 (6.1%)	84 (22.4%)	2.28
2	Provider at the nearest health facility examine thoroughly	248 (66.1%)	48 (12.8%)	79 (21.1%)	2.32
3	Providers at the nearest health facility have the motivation to help clients	319 (85.1%)	19 (5.1%)	37 (9.9%)	2.07
4	Providers at the nearest health facility take informed consent before any obstetric examination	204 (54.4%)	18 (4.8%)	153 (40.8%)	2.66
5	Health professionals provides timely follow-up during labor	153 (40.8%)	18 (4.8%)	204 (54.4%)	3.01
6	Health providers at the nearest health facility listen carefully to clients concerns during labor and delivery	110 (29.3%)	63 (16.8%)	202 (53.9%)	3.18
7	Health providers at the nearest health facility provide encouragement during labor and delivery	243 (64.8%)	36 (9.6%)	96 (25.6%)	2.40
8	Health providers at the nearest health facility are capable of handling birth-related complications	282 (75.2%)	6 (1.6%)	87 (23.2%)	2.41
9	Health providers at the nearest health facility provide full information regarding your health and baby during the labor process	171 (45.6%)	75 (20.0%)	129 (34.4%)	2.80
10	Health providers at the nearest health facility allow support persons including husband during labor	218 (58.1%)	21 (5.6%)	136 (36.3%)	2.58
11	Health providers at the nearest health facility recommends care to the next level early before complication arise	296 (78.9%)	19 (5.1%)	60 (16.0%)	2.35

### Obstetric related history for respondents among laboring mothers at Dilla universality hospital, Dilla, Ethiopia, 2021

About half (183, 48.8%) of the respondents had more than three antenatal care visits. About 123 (54.91%) reported having experienced obstetric-related complications during their current pregnancy. In terms of parity, approximately 180 (48% of the mothers) were primiparous, while 195 (52% were multiparous) (Table 4).

**Table 4:** Obstetric characteristics of the respondents at DURH, SNNPR, Gedeo Zone, Ethiopia, 2021

Obstetrics characteristics	Self-referred		Frequency (%)
	Yes (%)	No (%)	
*ANC visit*			
No visit	72	20	92 (24.5)
1–2 Visit	77	23	100( 26.7)
> 3 Visit	97	86	183 (48.8)
*Complication on current pregnancy*			
No	111	48	159 (42.4)
Yes	135	81	216 (57.6)
*First-time pregnancy*			
No	132	63	195 (52.0)
Yes	114	66	180 (48.0)

### Factors associated with self-referral among laboring mothers’ at Dilla University Referral Hospital, Gedeo Zone, Dilla, Ethiopia, 2021

According to bivariate analysis, those variables that have a *P*-value ≤ 0.25 were regressed against the dependent variable in multivariable logistic regression. As shown in Table 5 below, in multivariable logistic regression, educational status, mothers' monthly income, time to reach nearby health facilities, information about the referral system, medicine availability at the NHF, equipment and supplies at the NHF, antenatal care visit, complications during pregnancy, first-time pregnancy, and mothers' perception of HCP at the NHF were regressed against the dependent variable. However, in a multivariable logistic regression analysis, the following factors were significantly associated with self-referral: time to reach nearby health facilities; mothers' perception of health care providers (HCP) at the NHF; equipment and supplies at the NHF; and antenatal care visit.

**Table 5:** Bivariate and Multivariable analysis for self-referral among laboring mothers at DURH, Gedeo Zone, Ethiopia, 2021

Covariate	Self-referral		COR [95% CI]	AOR [95% CI]		*P* value
	Yes	No				
*Educational status*						
No education	25	9	1	1		
Primary	154	81	0.68 (.30, 1.53)	0.83 (.34, 2.03)		0.68
Secondary	55	28	0.70 (.29, 1.71)	0.85 (.31, 2.34)		0.75
Collage or above	12	11	0.39 (.12, 1.20) *	0.45 (.11, 1.77)		0.25
*Mothers monthly income*						
< 500 ETB	92	56	1	1		
501–1000 ETB	88	44	1.22 (.74, 1.9)	1.13 (.64, 2.00)		0.65
> 1000 ETB	66	29	1.39 (.80, 2.40)*	1.47 (.73, 2.94)		0.272
*Time to reach nearby health facilities*						
< 30 Minutes	100	68	1	1		
≥ 30 Minutes	146	61	1.63 (1.06, 2.49)*	1.74 (1.08,2.81)**		0.02
*Information regarding referral system*						
No	153	72	1	1		
Yes	93	57	0.76 (.49, 1.18)*	0.68 (.41, 1.10)		0.11
*Medicine availability at the NHF*						
No	135	54	1.68 (1.09, 2.59) *	1.75 (1.08, 2.82)**		0.02
Yes	111	75	1	1		
*Equipment and supplies at the NHF*						
No	115	42	1.82 (1.16, 2.83) *	1.70 (1.03, 2.78)**		0.03
Yes	131	87	1	1		
*Antenatal care visit*						
No visit	72	20	1	1		
1–2 Visit	77	23	0.93 (.47, 1.83)	0.75 (.36, 1.57)		0.45
> 3 Visit	97	86	0.31 (.17, .55)*	0.29 (.15, .55)**		0.00
*Complication during pregnancy*						
No	111	48	1	1		
Yes	135	81	.72 (.46, 1.11)*	0.64 (.39, 1.04)		0.07
*First-time pregnancy*						
No	132	63	1	1		
Yes	114	66	0.82 (.53, 1.26)*	0.83 (.519, 1.34)		0.45
*Mothers perception for HCP at NHF*						
Good	89	79	1	1		
Poor	157	50	2.79 (1.79, 4.33)*	2.96 (1.83, 4.79)**		0.00

“*”—Significant in bivariate level, “**”—significant multivariable level. NHF—Nearby health facility, HCP—health care provider working nearby health facility

Controled for the following variables; Residence, Age group, Marital status, Occupational status, Family size, House ownership, Adequate human resource, Laboratory service,Clean water and electricity, and Adequate private room

The odds of self-referral were 1.74 times higher among labouring women who travelled ≥ 30 min to the nearest health facility compared to those who travelled ≤ 30 min to the nearest health facility [95% CI: AOR 1.74 (1.08, 2.80) *p*-value = 0.02].

Mothers with poor perception of the technical competence of health care providers working at the nearest health facilities had 2.96 times the odds of self-referral than those with good perception [95% CI: AOR; 2.96 (1.83, 4.79) *p*-value 0.00].

The odds of self-referral were 1.70 higher among mothers who did not have confidence in the nearest health facilities having adequate equipment and supplies compared to those who did have confidence in the nearest health facilities having adequate equipment and supplies [95% CI: AOR; 1.70 (1.03, 2.78) *P*-value = 0.03].

In terms of the availability of medicine supply at the nearest health facilities, the odds of self-referral among mothers who did not have confidence in the nearest health facilities having adequate medicine supply were 1.75 times higher than those who did have confidence in the nearest health facilities having adequate medicine supply [95% CI: AOR; 1.75 (1.08, 2.82) *P*-value = 0.02].

In addition, mothers who had three or more antenatal care visits were nearly 70.5 percent less likely to self-refer than those who had none [95% CI AOR; 0.29 (0.15, 0.55) *P*-value 0.00]. (Table 5).

## Discussion

The magnitude of self-referral (bypassers of the first level facilities) among laboring mothers at Dilla University Referral Hospital was 246 (65.6%) with 95% CI (0.60–0.70). Within different settings, the magnitude of self-referral (bypassing) varies. This result is higher than that of a study done in Uganda, where 204 (29%) of women bypassed their closest facility to give birth in the district hospital, but it is lower than that of a study done in Ghana, where 629 (32.3%) of women reported skipping their closest facilities for primary healthcare. Since the study was conducted using community-based studies in the two countries, the variance may be due to the large sample included in the study [29, 30].

Our results, however, were nearly in agreement with a study conducted in Nepal, which revealed that 70.2% of expectant women chose to forego birth centers. The comparable result with the Nepali study can be attributed to the study population's similarity, which is maternal health care users[21]. In contrast to two similar studies conducted in India, we find that 280 (38.9%) and 357 (37.7%) mothers self-refer to higher level facilities. The figures are still higher than the findings of our study, which could be due to a difference in sample size because the data is gathered from multiple sources, both in the community and in health facilities [23, 31].

Considering the differences in the study populations and settings compared to our findings, the result was similar to studies conducted at Debretabor Regional Hospital and Western Ethiopian, which showed a self-referral rate of 63.9% and 82.1%, respectively [10, 17, 25]. similarly, our findings were comparable with a study conducted in South Ethiopia at Negist Eleni Memorial Hospital, which discovered that 67% of postnatal mothers were bypasers of the proximity health facilities [19]. Our justification for this consistency could be attributed to the similarity of the governing health care systems across the country.

Patients can easily travel to and visit health facilities if located in reasonable distance and have easy access to transportation. Our finding shows the odds of self-refral were 1.74 times more likely for mothers who took ≥ 30 min to walk to reach the nearest health facility. Study in china reveled that longer travel time to primary facilities compared to higher-tier facilities increases the likelihood of bypassing [32]. Similarly in Northern Ethiopia they found that the less likely mothers were to use the nearest health facilities for skilled delivery, the longer it took to reach the nearest health facilities (> 30 min by walk) [9]. According to a study in Adiss Ababa, women tend to avoid the nearest health facilities located within 13.02 km when compared to those living further away [19]. Similarly, in Western Ethiopia, they revealed that the inconvenience of transportation to reach facilities due to a remote geographic location was significantly associated with self-referral [17]. Furthermore, maternal mortality due to direct obstetric causes is strongly related to the long distance to the nearest health facilities and residences in rural Tanzania [33].

When compared to those who never attend their ANC visit, mothers who had more than three ANC visits were 70.5% less likely to self-referral. These results are in line with that of the Negist Eleni Memorial Hospital study, which found that women who received antenatal care in health centers were less likely to have bypass [19]. Our results were in agreement with a cohort study carried out in Nepal, which revealed that frequent (4 visits) antenatal care visits were a protective factor for skipping primary health care units [21]. This disparity could be attributed to the quality of ANC services provided at nearby health facilities, which influences them to follow the proper pathway of the existing referral system.

On the contrary, a cross-sectional study in Nepal's Chitwan shows ANC vist doesn't play a role in bypassing birthing facilities [34]. However, a study conducted in India and Uganda revealed mothers who had at least three ANC visits bypassed primary health care units [23, 29]. This may be due to a difference in the study design employed and confounding variables, or the mothers awareness may have increased about complications during their current pregnancy through frequent ANC visits that needed care not available at their nearest facility, which might have also influenced the mother’s decision to self-refer.

The odds of self-referral among mothers was 2.962 more likely among those believed that the provider at the nearby medical facility did not have a high level of technical competence. Our finding was in line with a study done in Tanzania that found bypassing was 2.7 times more likely for women who trusted the medical staff at the facility the mothers chose for delivery [22]. Studies conducted in Nigeria, Nepal, and India have revealed a similar pattern, demonstrating that the major causes of bypassing decisions were a lack of medical knowledge necessary to provide for them and a lack of competent staff in primary health facilities, and that this event decreased as clinicians' competency increased [3, 34, 35]. Similarly, Participants in a cross-sectional study in the capital city and a FGD conducted in four regions of Ethiopia, indicated that they would bypass the primary health care units because "these facilities often lack competent staff." Poor staff motivation and poor client perception have also been noted as obstacles to obstetric referra [5, 6]. This study argues the lack of current training on knowledge and competency in the lower health care system may have contributed to the mother's negative perception.

Mothers who were unsure about the availability of supplies and equipment at nearby healthcare facilities were 1.75 times more likely to self-refer and supplies at nearby medical facilities, and it was 1.70 times more likely for mothers who were unsure about their ability to obtain prescribed medications from those facilities. Related study in Ethiopia has shown that self-referral to a referral hospital is strongly associated with receiving prescribed medications, suplies and anticipating access to improved laboratory tests [6, 10, 17, 25]. Studies conducted in South Africa, Nigeria, Tanzania, and Nepal also revealed that pregnant women's self-referral to higher-level facilities was primarily motivated by PHCU's lack of drugs and medicine, equipment and supplies [28, 31, 32, 36]. This confirms that if primary health care units have a good reputation for meeting basic needs, they are more likely to be used, which could otherwise be the primary reason for self-referral by mothers.

### Limitation

This study was not addresed the mother’s actual health experience at the nearest health facilities. Primary health care facilities should be evaluated in a direct and objective manner. The mothers' opinions alone are insufficient to reveal the true characteristics of the primary health care facility.

## Conclusion and recommendations

According to the findings of this study, the magnitude of self-referral at referral hospitals was high, based on the assumption that the primary level of health care facilities was supposed to be the first point of contact for all patients. The time to reach the nearest health facility and the mothers' poor perception of the health care provider's technical competence, the availability of equipment and supplies, and medicine availability at the nearest health facility are the main factors that positively influence laboring mothers to self-refer. Mothers who had more frequent ANC visits, on the other hand, were found to be protective factors against self-referral. Therefore, the Gedeo zone health bureau should construct primary health care facilities at a reasonable distance for easy access and utilization.The zonal health bureau should continue to work on getting medical supplies and labaratory services to primary care facilities. Primary health care units (PHCU) should be encouraged to increase their ANC visits in order to retain their local clients.Furthermore, primary health care providers should strive to correct the mother's perception by improving their skills and providing the best care possible.

## Data Availability

The datasets generated and /or analyzed during the current study are not publicly available due to preserving participant anonymity but are available from the corresponding author upon request through the email address (wondwosenm955@gmail.com).

## Abbreviations

DURH: Dilla University Referral Hospital
HCP: Health care providers
NHF: Nearby health facilities
PHCU: Primary health care units
SNNPR: Southern Nations, Nationalities, and Peoples' Region
